# Neck of the 5th metacarpal head retrograde screwing with a headless screw after a recurrent fracture on osteosynthesis pins: a case report

**DOI:** 10.1016/j.ijscr.2025.111418

**Published:** 2025-05-17

**Authors:** Cyril Lemé, Matthieu Peras, Bernard de Geoffroy, Lilian Gaubert, Olivier Barbier

**Affiliations:** a Service de Chirurgie Orthopédique et Traumatologique, HIA Sainte-Anne, 2 boulevard Sainte-Anne, 83000 Toulon, France; b IULS-University Institute for Locomotion and Sports, Pasteur 2 Hospital, Unité de Recherche Clinique Côte d'Azur (UR2CA), Hôpital Pasteur II, 30, voie Romaine, 06000 Nice, France

**Keywords:** Traumatology, Hand surgery, Osteosynthesis, Case report

## Abstract

**Introduction and importance:**

This paper reports the technique we used to treat a recurrent fracture of the neck of the 5th metacarpal with bending of the pin that was still present in the patient bone.

**Case presentation:**

A 26-year-old soldier presents to the emergency department following a direct trauma on the right hand. This patient had already presented a similar trauma one year ago with a fracture of the 5th metacarpal neck treated by intramedullary pins according to Foucher technique's. X-rays showed a recurrent fracture of the 5th metacarpal neck with pins bended. It was decided to remove the material with a change of osteosynthesis technique by intramedullary screwing with a headless screw. This allowed a return to work and activities without restriction 6 weeks after surgery.

**Clinical discussion:**

to our knowledge this is the first publication dealing with management of recurrent fracture of 5th metacarpal neck. This technique provides excellent outcome and a low risk of complication.

**Conclusion:**

This simple and original technique for revision osteosynthesis of a fracture the 5th metacarpal neck gives good short-term results and is interesting from a practical and functional point of view in the military patients.

## Highlights

• We received in the E.D a soldier with an iterative fracture of the 5th metacarpe.
• X-rays showed that his osteosynthesis pin was bended.
• We performed a one-stage surgery with placement of a centro medullary screw to treat and prevent a new fracture.

## 1 Introduction

Fifth metacarpal neck fractures account for almost a quarter of hand fractures [1]. Gold standard technique when surgical treatment is chosen is pinning according to Foucher technique's [2]. Retrograde intramedullary screwing of these fractures was described in 2010 by Boulton with excellent clinical results and above all the absence of postoperative immobilization and the need to remove the material [3].

Literature is very poor concerning the management of recurrent fractures of the 5th metacarpal. To our knowledge, there is no description of a technique for revision osteosynthesis of the 5th metacarpal neck's fracture with osteosynthesis pins in place.

**Fig. 1:**
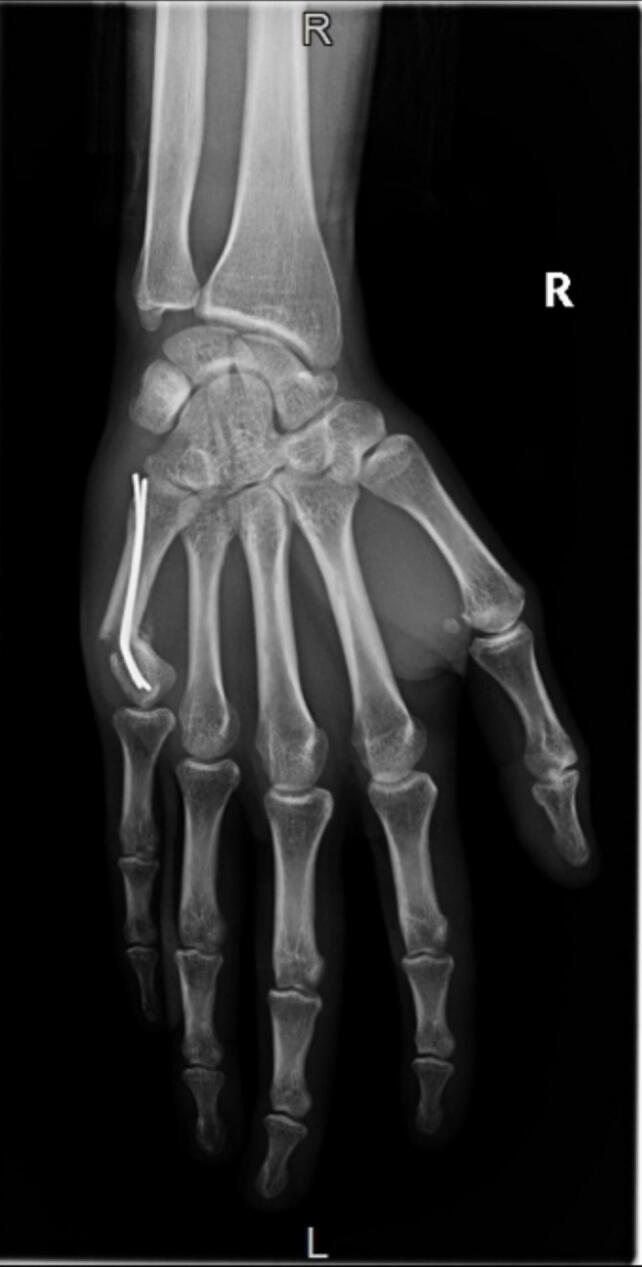
Preoperative X-ray showing bended pins in the fracture.

This article reports the technique that we performed for a case of a recurrent fracture of the 5th metacarpal neck in a soldier who already had two osteosynthesis pins placed during the first injury (Fig. 1), in a military teaching hospital.

This work has been reported in line with the SCARE criteria [4].

## 2 Patient information

A 26-year-old (non-smoker and right-handed) soldier presents to the emergency department following a direct on the right hand. He had already presented 12 months ago a similar trauma with a closed fracture of the neck of fifth metacarpal (M5). He has been treated by osteosynthesis with two pins according to Foucher technique's. X-rays showed a recurrent fracture of M5 neck's with bending of the two osteosynthesis pins at 40° in place (Fig. 1), with a major clinodactyly. After discussing the case in traumatology staff, we decided to remove pins and perform a retrograde osteosynthesis by centromedullary screwing with a headless screw.

## 3 Presentation of the technique

**Fig. 2:**
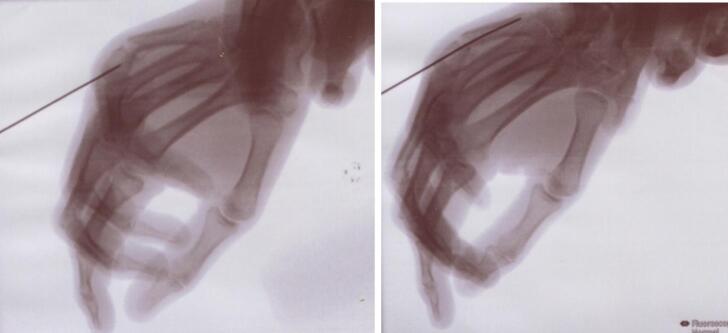
Fracture reduction with the S.B.I® pin.

**Fig. 3:**
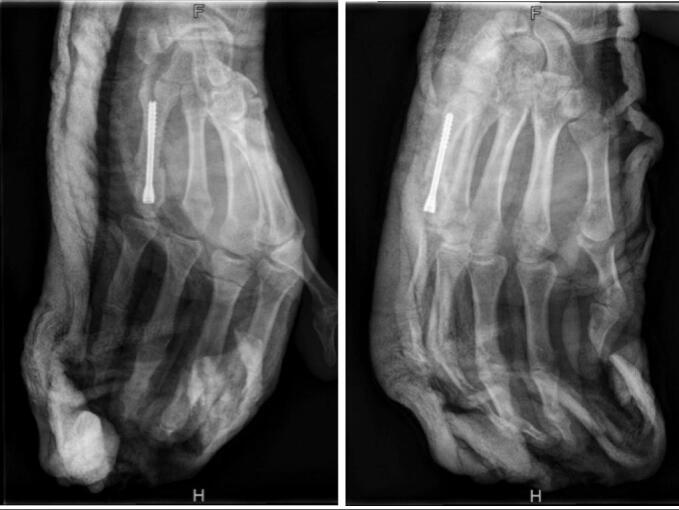
Postoperative X-rays.

We operated on the patient one day after trauma. Surgery was performed by an experimented hand surgeon. The patient was installed in the supine position. The intervention takes place with a C-Arm control. First, the pins are removed. A mini-approach on the head of M5 is then performed to be able to protect the extensor with a Senn-Miller retractor. The fracture is then reduced by joystick using the S.B.I® (Small Bone Innovation®, Morrosville, Pennsylvania, United-States) ancillary pin, introduce with the maximal flexion of the metacarpophalangeal joint, and fixed with a S.B.I® screw diameter 4.0 mm (Fig. 2, Fig. 3). Skin is closed with non-absorbable sutures. The patient was immobilized with a metacarpal cast with a syndactyly of the 4th and 5th fingers, rehabilitation is started immediately.

The operative technique was very easy, and we didn't have any challenge or unexpecting finding during the operation.

**Fig. 4:**
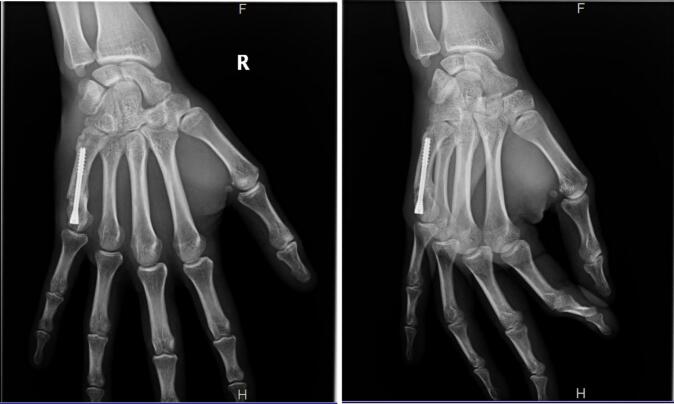
X-rays 6 weeks post-operative.

The patient was seen on a consultation at 15 days, 6 weeks, 3 months and 1 year after his surgery. Bone consolidation was acquired at 6 weeks (Fig. 4). He recovered complete and painless mobility of the 5th ray of the right hand at 6 weeks and resumed his military activity at the same position without restriction of aptitude.

## 4 Discussion

We did not find in the literature any previous case of a recurrent fracture of the 5th metacarpal with the presence of bended pins.

We opted for this surgical solution because of the patient's age, his functional demands due to his profession, and because of the solidity of this new osteosynthesis material. Moreover, in the case of a new trauma on this hand, the entire metacarpal is now “armed” and prevents any new iterative fracture. Finally, the headless screw authorizes to not remove the material, which could be a new unavailability in service for this soldier.

The technique described by Boulton theoretically does not require postoperative immobilization and a return to sports activities once the wound is closed [5]. We chose immobilization because it was a revision surgery, we wanted to make sure the patient was compliant, and we wanted to ensure optimal bone consolidation as soon as possible.

We can be criticized because of the risk of injury to the extensor and cartilage damage to the metacarpal head when performing this technique. This risk is theoretical with studies showing that lesions of the extensors are rarer than during the use of percutaneous techniques and that cartilage lesions are minimal, taking on average only 20% of the cartilage surface on a non-weight-bearing joint and therefore non-symptomatic [6]. Joint stiffness and material failure are also described but this kind of complication represents less than 2,5% of all intramedullary screwing [7].

## 5 Conclusion

This simple and original technique of revision osteosynthesis of the 5th metacarpal neck's fracture gives good short-term results and is interesting from a practical point of view in the military patient.

## Author contribution

Cyril Lemé: wrote and corrected manuscript.

Matthieu Peras: collected iconnographie.

Bernard de Geoffroy: operated on patient.

Lilian Gaubert: operated on patient.

Olivier Barbier: supervised work.

## Informed consent

Patient has given consent for publication.

## Ethical approval

Publication has been validated by local ethic committee of Hospital Sainte-Anne on the 09th September 2023.

## Guarantor

Matthieu Peras is the Guarantor for this work.

## Research registration number

Not applicable.

## Funding

Not applicable.

## Conflict of interest statement

Author don't declare any conflict of interest that could influence on this work.
